# Efficacy of Platelet-Rich Plasma Therapy in Oral Lichen Planus: A Systematic Review

**DOI:** 10.3390/medicina59040746

**Published:** 2023-04-11

**Authors:** Shyamkumar Sriram, Shamimul Hasan, Abdullah Alqarni, Tanveer Alam, Sultan Mohammed Kaleem, Shahid Aziz, Humayoun Khan Durrani, Muhammed Ajmal, Ali Azhar Dawasaz, Shazina Saeed

**Affiliations:** 1 Department of Social and Public Health, Ohio University, Athens, OH 45701, USA; ssriram@ohio.edu; 2 Department of Oral Medicine and Radiology, Faculty of Dentistry, Jamia Millia Islamia, New Delhi 110025, India; 3 Department of Diagnostic Dental Sciences and Oral Biology, College of Dentistry, King Khalid University, Abha 62529, Saudi Arabia; aawan@kku.edu.sa (A.A.); talam@kku.edu.sa (T.A.); skalem@kku.edu.sa (S.M.K.); drmajmal@kku.edu.sa (M.A.); adwasaz@kku.edu.sa (A.A.D.); 4 Department of Medicine, College of Medicine, King Khalid University, Abha 62529, Saudi Arabia; saziz@kku.edu.sa (S.A.); hkdurrani@kku.edu.sa (H.K.D.); 5 Amity Institute of Public Health & Hospital Administration, Amity University, Noida 201303, India

**Keywords:** autoimmune, oral lichen planus, oral potentially malignant disorder, platelet-rich plasma, treatment

## Abstract

*Background and Objectives*: Oral lichen planus (OLP) is an autoimmune, mucocutaneous, oral potentially malignant disorder (OPMD), which characteristically manifests with chronic, recalcitrant lesions, with frequent flare-ups and remissions. The precise etiopathogenesis of OLP is still debatable, although it is believed to be a T-cell-mediated disorder of an unidentified antigen. Despite the availability of various treatments, no cure for OLP exists due to its recalcitrant nature and idiopathic etiology. Platelet-rich plasma (PRP) has antioxidant, anti-inflammatory, and immunomodulatory properties, in addition to its regulatory action on keratinocyte differentiation and proliferation. These salient properties substantiate the possible role of PRP in the treatment of OLP. Our systematic review focuses on assessing the therapeutic potential of PRP as a treatment modality in OLP. *Materials and Methods*: We conducted a detailed literature search for studies assessing PRP as a therapeutic regimen in OLP, using the Google Scholar and PubMed/MEDLINE search engines. The search was limited to studies published from January 2000 to January 2023 and included a combination of Medical Subject Heading (MeSH) terms. ROBVIS analysis was carried out for the assessment of publication bias. Descriptive statistics were performed using Microsoft Excel. *Results*: This systematic review included five articles that met the inclusion criteria. Most of the included studies demonstrated that PRP treatment considerably ameliorated both objective and subjective symptoms in OLP subjects, with comparable efficacy to the standard corticosteroid treatment. Further, PRP therapy offers the added benefit of minimal adverse effects and recurrences. *Conclusion*: This systematic review suggests that PRP has significant therapeutic potential for treating OLP. However, further research with larger sample sizes is imperative to corroborate these findings.

## 1. Introduction

Lichen planus (LP) is a chronic inflammatory disorder of the skin, mucosa (oral and genital mucous membranes), skin appendages, and nails. The most common form of mucosal LP is oral LP (OLP), which can appear as exclusive oral lesions or may be accompanied by cutaneous, nail, scalp, or other mucosal lesions (genital, gastrointestinal, ocular, and laryngeal) [1].

Around 60–70% of cutaneous LP patients may present with oral lesions, while isolated oral lesions may be seen in 20–30% of patients [2]. Cutaneous LP is usually self-healing and non-pruritic, with 85% of patients showing complete resolution within 18 months [3]. OLP represents the mucosal counterpart of skin LP [4], is often chronic, and is recalcitrant to therapy [3]. The malignant potential of oral lesions is often ascribed to the accompanying morbidity [5,6].

Oral lesions are usually bilaterally symmetric and typified as white or gray-white papular lesions surrounded by linear, circular interlacing striae (Whickham’s striae) [5]. OLP can be categorized into reticular, papular, plaque-like, erosive, atrophic, and bullous forms [7]. A recent classification identified three forms of OLP, namely, reticular, atrophic, and erosive or ulcerative forms [8,9]. Reticular OLP lesions are usually asymptomatic [10]; however, the atrophic and erosive or ulcerative lesions may cause mild to severe itching and burning sensations. This may hinder functional activities, such as chewing, swallowing, and speech, thus leading to impaired oral health and quality of life [11].

The global pooled prevalence of OLP is 1.01% and displays noticeable geographical variation. The highest prevalence was documented in South-Central America (1.74%) and the lowest in India (0.49%), with individuals over 40 years of age showing almost 3.5-times higher prevalence of OLP lesions [12]. OLP primarily affects females over 40 years of age [13].

The cause of OLP is not yet fully understood but is considered a chronic autoimmune oral mucosal disorder mediated by an antigen-specific response that provokes T cells after a non-specific response of mast cell degranulation [14,15,16].

The diagnostic criteria for OLP are a topic of much debate due to the lack of specific guidelines [13]. The WHO first published clinical and histopathologic criteria for OLP diagnosis in 1978, which did not clarify whether epithelial dysplasia should be distinguished or excluded from the OLP diagnosis [17]. The WHO criteria were later modified by Van der Meiji and van der Waal in 2003 to confirm OLP diagnosis in the absence of epithelial dysplasia and attempted to eliminate lichenoid dysplasia from OLP [18]. The American Academy of Oral and Maxillofacial Pathology advocated diagnostic criteria for OLP in 2016, emphasizing the importance of clinicopathologic correlations for a confirmatory diagnosis [7].

Recent studies supported the use of clinicopathological attributes for a conclusive diagnosis, thus highlighting the need for comprehensive documentation of all demographic, medical, and environmental variables in OLP cases [19].

OLP is regarded as an oral potentially malignant disorder (OPMD), with a controversial malignant transformation rate, owing mainly to its restrictive diagnostic criteria [20]. However, recent studies have documented a malignant transformation rate ranging from 0.44% to 2.58% [19,21,22,23,24,25,26], with an increased malignant transformation risk in cases of erosive and/or atrophic lesions [19,21,24,25,26], tongue lesions [19,24,25], greater intake of alcohol/tobacco [19,24,25], accompanying hepatitis C virus infection [19,24,25,26], and elderly females in an age range of 60–70 years [21,26]. Therefore, annual monitoring is recommended to detect early malignant lesions, and this should be performed by oral medicine specialists [1,21].

The treatment of OLP should aim to achieve specific goals, such as reducing atrophic and ulcerative lesions, alleviating symptoms, and potentially reducing the risk of malignant transformation [11,14,16,27]. Most published reviews agree that only symptomatic or erosive/ulcerative forms of OLP require treatment, while asymptomatic reticular lesions require constant follow-up. It is also advisable to eliminate all the risk factors in the oral cavity, such as occlusal disharmony and poor oral hygiene, and to quit deleterious habits (smoking and alcohol) [11].

Topical steroid therapy is recommended for mild–moderately symptomatic localized OLP lesions, with minimal systemic absorption and undesirable effects in the adrenal gland [24,28]. Systemic steroids are only used in situations where topical treatments have proven to be ineffective. They are also used for recalcitrant erosive/erythematous lesions, or in cases where there are widespread OLP lesions accompanied by cutaneous, genital, and scalp lesions [13,24].

A recent meta-analysis conducted on 55 randomized controlled trials showed that topical corticosteroids are the most effective treatment for OLP [29].

Other treatment options include topical calcineurin inhibitors (pimecrolimus, tacrolimus, and cyclosporin); systemic immunosuppressants (mycophenolate mofetil, methotrexate, azathioprine, and dapsone); retinoids (tretinoin, isotretinoin, and tazarotene); immunostimulants (thalidomide and levamisole); biological agents (TNF-α inhibitors and BCG-PSN); nutraceuticals (aloe vera, lycopene, purslane, ignatia, curcumin, and quercetin); and novel therapies, such as amlexanox, hyaluronic acid, and amitriptyline. Low-level laser therapy (LLLT), photodynamic therapy (PDT), cryotherapy with nitrous oxide gas, and ozone therapy have also been suggested for patients with symptomatic OLP [1,16,24,27].

The therapeutic efficacy of calcineurin inhibitors (pimecrolimus, tacrolimus, and cyclosporin) in OLP may be attributed to their ability to bind FK506-binding protein to T-cell cytoplasmic proteins, thereby inhibiting the transcription and synthesis of various proinflammatory cytokines [30].

Transient burning or stinging sensations during drug application were the most commonly documented local adverse effects of tacrolimus. Additionally, some users experienced altered taste alteration and sensitivity to hot, cold, or spicy foods. Nevertheless, these adverse effects were generally minor and transient and tended to improve or resolve over time [31]. Long-term use of tacrolimus may augment the onset of mucosal superinfections and the malignant transformation risk [32]; thus, the prolonged use of tacrolimus should be averted, and stringent long-term monitoring of OLP patients should be carried out [31].

In a recent randomized controlled clinical trial, the therapeutic efficacy of tacrolimus (0.1%) ointment was compared to an anti-inflammatory mouthwash containing hyaluronic acid, calcium hydroxide, oligomeric proanthocyanidins, and umbelliferone. Both therapies exhibited significant effectiveness in managing OLP; however, after a 3-month follow-up, tacrolimus was found to be more successful in ameliorating OLP signs and symptoms compared to the anti-inflammatory mouthwash [33]’.

The published literature has demonstrated that topical cyclosporin is equally efficacious to topical steroids as OLP treatment modality [30]. However, systemic cyclosporine is not recommended for routine OLP therapy, owing to the associated adverse effects (nephrotoxicity, hypertension, and gingival hyperplasia) [1].

Mycophenolate mofetil (MMF) exerts strong cytostatic action, primarily on the lymphocytes. Common side effects of MMF use include gastrointestinal problems and reduced peripheral leukocytes. However, currently, there is insufficient evidence to advocate the routine use of MMF for OLP treatment [34].

Azathioprine (AZA), an anti-metabolite, hinders purine synthesis and results in decreased T- and B-lymphocyte proliferation. Along with its immunosuppressive effects, AZA also exhibits salient anti-inflammatory properties. However, prudent systemic use of AZA in diffuse OLP cases is warranted due to the associated life-threatening adverse effects (liver dysregulation, pancytopenia, and immunosuppression) [28].

Methotrexate (MTX), a folate antimetabolite, acts by inhibiting DNA synthesis, repair, and cell replication. Patients receiving MTX may experience dose-related adverse effects, such as skin rash, stomatitis, and gastrointestinal problems [1]. Studies have documented that MTX may be regarded as a first-line option for patients with moderate to severe OLP, either as a standalone treatment or in conjunction with topical triamcinolone [35].

Dapsone is an antileprotic drug, generally used in conjunction with Rifampicin and Clofazimine. However, there is a dearth of published literature highlighting the therapeutic role of dapsone in OLP, and only two case reports have documented the therapeutic efficacy of dapsone in OLP [36,37]. The associated hematological adverse effects (methemoglobinemia and hemolytic anemia) as well as the availability of several alternate therapies have limited the routine use of dapsone in OLP patients [1].

Retinoids are vitamin A derivatives and can be administered topically or orally to treat OLP [28]. Topical retinoids (tretinoin, isotretinoin, and fenretinide gels) are effective in diminishing reticular and plaque lesions; however, relapse is often observed upon discontinuing the treatment [38]. On the other hand, the use of systemic retinoids is limited due to the potential side effects, such as cheilitis, liver damage, and teratogenicity [4,38].

Thalidomide inhibits tumor necrosis factor alpha, a proinflammatory cytokine involved in the pathogenesis of OLP. Additionally, thalidomide has been observed to enhance the function of T cells, macrophages, and NK cells. It affects apoptosis by lowering the anti-apoptotic Bcl-2 levels and making lesional cells more responsive to apoptosis initiated by Fas [16]. In a randomized controlled trial, topical thalidomide was considered as efficient as dexamethasone in the management of erosive oral lichen planus [39]. However, thalidomide’s potential for severe adverse effects, such as teratogenicity and peripheral neuropathy, restricts its use [40].

Levamisole, an anthelminthic drug, also exerts significant immunomodulatory effects. It functions by boosting the activity of interleukins, interferons, and T-cell-mediated immunity [16]. A prospective study recommended the use of levamisole in conjunction with low doses of systemic corticosteroids as a treatment strategy for severe erosive OLP [41].

Aloe vera exhibits an anti-inflammatory effect, thereby inhibiting the cyclo-oxygenase pathway and the consequent decreased prostaglandin E2 production. It further impedes the release of histamine and leukotriene from mast cells that are triggered by antigen–antibody reactions, a critical element in OLP pathogenesis [1,15,16]. However, there are insufficient data to arrive at a definitive conclusion on the substitution of aloe vera for conventional OLP treatment [16].

Amlexanox is a topical anti-inflammatory agent (used as 5% oral paste) to treat recurrent aphthous stomatitis. It acts by inhibiting the synthesis and release of histamine, leukotrienes, and TNF alpha from mast cells, mononuclear cells, and neutrophils [42]. A randomized clinical trial demonstrated comparable therapeutic effectiveness of 5% amlexanox paste with that of 0.043% dexamethasone paste in OLP [43]. Generally, 5% amlexanox paste does not cause adverse effects, although a few patients have reported experiencing mild transient tingling sensations and a metallic taste [42].

Hyaluronic acid (HA) plays a key role in several biological processes, such as cell signaling, cell proliferation, gene expression regulation, morphogenesis, matrix organization, lubrication, tissue hydration, and wound healing. One of the greatest advantages of hyaluronic acid is its safety profile, as it can be safely used in all patients, including infants and pregnant females. Additionally, it can be used in all grades of oral ulceration [14]. A study by Yousef et al. concluded that topical HA (0.2%) demonstrated higher efficacy in diminishing OLP symptoms as compared to topical corticosteroids [44].

Low-level laser therapy (LLLT), also referred to as photobiomodulation, is a non-pharmacological, non-invasive treatment protocol. It has potent anti-inflammatory, analgesic, bio-stimulating, and immunomodulatory properties, with minimal adverse effects [45]. A systematic review conducted by Al-Maweri et al. established the therapeutic efficacy of LLLT in symptomatic OLP cases, and LLLT may be employed as a replacement for corticosteroids [46].

Photodynamic therapy (PDT) employs a photosensitizing dye (methylene blue) activated by laser light for a specific wavelength. The active agent kills the target cells via potent oxidizers, causing cell damage, protein inactivation, and membrane lysis. The precise mode of action of PDT in OLP is still obscure, but the proposed recommendations emphasize that it may exhibit immunomodulatory effects by initiating apoptosis in the hyperproliferating inflammatory cells of OLP [1,16]. According to He Y et al., PDT exhibits comparable therapeutic efficacy to topical corticosteroids and may be employed in recalcitrant cases or cases where steroids are contraindicated. PDT may be used as a viable and effective treatment strategy in OLP [47].

Despite the availability of various treatments, no cure for OLP exists due to its recalcitrant nature and idiopathic etiology [14,16].

Moreover, different preparations and classes of topical steroids vary in efficacy and cost, not all patients respond favorably to steroids, and several local and systemic adverse effects may limit extended steroid use [48]. Topical steroids lack adherence to the mucosa for a sufficient length of time [49]. The lack of potency of steroids in OLP patients could be due to several factors, including an inadequate selection of the vehicle and inappropriate prescription dose, time, and/or frequency. However, despite an appropriate protocol, some lesions may not respond to topical treatment and necessitate alternate treatment protocols [50].

One potential alternative treatment option for OLP is platelet-rich plasma (PRP), which refers to human platelet concentrates derived from a patient’s blood (autologous), containing 3- to 5-times more platelets than the normal concentration found in whole blood. Another distinctive feature of PRP is that it is an autologous product, thus eliminating apprehensions regarding the risk of cross-contamination, disease dissemination, or immune reactions [51].

PRP contains bioactive molecules, such as growth factors, cytokines, and cell adhesion molecules. The biological justification for PRP use in regenerative medicine involves platelet degranulation, thus permitting the release of growth factors, amending the inflammatory reaction, and promoting cell proliferation and differentiation within the target tissue. PRP use has expanded considerably, encompassing many disciplines of medicine, including sports medicine, orthopedics, dermatology, cosmetic medicine, dentistry, maxillofacial surgery, and wound healing [52].

The therapeutic effects of autologous platelet concentrates have been demonstrated in various autoimmune diseases in the published literature. For instance, Huber et al. demonstrated that PRP had a beneficial effect on patients with Behcet’s disease and oral ulcers, resulting in a significant increase in T-regulator cells (Tregs) and stable anti-inflammatory cytokine activity [53]. A randomized controlled trial [54] and several case series [55,56] have also shown that PRP is effective in treating patients with rheumatoid arthritis. Additionally, PRP has demonstrated therapeutic efficacy in patients with vitiligo [57], psoriasis [58], and alopecia areata [59]. Promising results have also been reported in the treatment of genital lichen sclerosus [60] and scalp lichen planopilaris [61].

The ineffectiveness of conventional therapies for chronic autoimmune mucocutaneous disorders and the absence of preventive measures in wound healing have generated interest in the development of drugs/interventions that rely on specific biological mechanisms [62]. Previous studies have indicated that PRP can be therapeutically effective for patients with oral Pemphigus vulgaris [63,64].

OLP has been associated with impaired function in regulatory T lymphocytes, keratinocytes, and cell–matrix communication, as well as deficits in growth factors, such as transforming growth factor β (TGF-β) and fibronectin [65]. TGF-β1 plays a role in suppressing the immune response to self-antigens, and its deficiency makes the body more susceptible to the development of autoimmune diseases, such as OLP [4]. PRP also contains other growth factors, such as platelet-derived growth factor (PDGF), transforming growth factor beta (TGF-β), epithelial growth factor (EGF), insulin-like growth factor (IGF), vascular endothelial growth factor (VEGF), fibronectin, serotonin, dopamine, histamine, adenosine, and calcium, all of which have a variety of functions that promote cell differentiation, proliferation, and regeneration [66]. PDGF and TGF-β, in particular, have been shown to stimulate fibroblast proliferation and increase collagen production, while TGF-α and EGF can regulate the propagation and migration of keratinocytes, which leads to an increase in the thickness of the epidermis. PRP additionally enhances the expression of matrix metalloproteinases (MMPs), which regulate remodeling [60]. Thus, these anti-inflammatory, antioxidant, and immunomodulatory properties of PRP make it a promising therapy for OLP patients [67,68].

However, there is limited published literature on the therapeutic efficacy of PRP in OLP, with only a few case reports [69,70].

Therefore, this systematic review was conducted to establish and confirm the therapeutic role of PRP in OLP and to address any knowledge gaps that may help guide the formulation of new treatment guidelines for OLP.

## 2. Materials and Methods

The PRISMA (Preferred Reporting Items for Systematic literature reviews and Meta-Analyses) 2020 guidelines were followed for this systematic literature review.

### 2.1. Research Question

The systematic review utilized the PICO format to identify keywords related to the population, intervention, control, and outcomes for the search: (a) population—“oral lichen planus (OLP)”; (b) intervention/exposure—“Platelet-rich plasma”; (c) control—“OLP patients treated with corticosteroid therapy or other treatment modalities”; and (d) outcome—“efficacy evaluation”.

The research aim of the review was “to evaluate the therapeutic role of platelet-rich plasma in OLP patients”.

### 2.2. Inclusion Criteria

The inclusion criteria for the systematic review were as follows: (a) studies on human subjects with PRP-treated OLP; (b) articles in the English language published between January 2000 and January 2023; (c) at least 10 study participants; and (d) studies evaluating the therapeutic effectiveness of PRP as an outcome measure.

### 2.3. Exclusion Criteria

The exclusion criteria were as follows: (a) studies evaluating the efficacy of plasma-rich fibrin in OLP patients; (b) studies on human subjects with cutaneous LP; (c) articles before January 2000 and published in languages other than English; (d) sample size < 10 subjects; (e) letter to editor, case reports, case series, and review articles.

### 2.4. Literature Search and Identification of Studies

The methodology for the systematic review was designed in adherence to the Preferred Reporting Items for Systematic Reviews and Meta-Analyses (PRISMA) 2020 guidelines to guarantee transparency, iteration, and comprehensive reporting. The PRISMA statement comprises a 27-item checklist that ensures these factors for systematic reviews [71]. A comprehensive literature search was conducted on the Google Scholar and PubMed/MEDLINE databases for studies that evaluated the therapeutic efficacy of PRP in OLP patients from January 2000 to January 2023 with the following Medical Subject Heading (MeSH) terms, “Oral lichen planus”, AND “platelet-rich plasma”. The search protocol was as follows: (“Lichen Planus, Oral” [Mesh] OR “Lichen Planus, therapy” [Mesh] AND “platelet-rich plasma” [Mesh] OR “first generation platelet concentrates”.

### 2.5. Study Selection

The titles and abstracts of the retrieved studies were evaluated methodologically by two authors (S.H. and S.S.), and the third author (S.K.S) resolved any disparity. The complete texts of potentially eligible studies were obtained and further evaluated for incorporation in the systematic review. In addition, the references from the included studies were manually searched to include any study that might have been missed during the initial search (A.A. and T.A.).

### 2.6. Outcome Parameters

The efficacy of the employed treatment protocols was evaluated by appraising the different objective and subjective outcome scoring systems used in the included studies. Objective symptoms, such as clinical appearance and severity of the lesions, and subjective symptoms, such as pain and burning sensations, were assessed using the Visual Analog Scale (VAS) and Numeric Rating Scale (NRS).

### 2.7. Data Extraction

The included articles were analyzed to extract the following details: names of author(s), publication year, country of study, study design, age and gender of the participants, sample size, the criteria used for OLP diagnosis, therapeutic regimens employed, the test of significance, and study outcome.

### 2.8. Risk of Bias Assessment

The National Institute for Health Research (NIHR) created an R package and a Shiny web app as part of the Doctoral Research Fellowship (DRF-2018-11-ST2-048) at the University of Bristol (UK) to evaluate the possibility of publication bias. The current 2020 version of the program was utilized for the analysis [72]. The six domains evaluated by the program include 1. randomization procedure, 2. recommended intervention, 3. missing outcome data, 4. outcome assessment, 5. selection of the reported results, and 6. overall assessment.

## 3. Results

Five eligible articles were finally considered for inclusion and were further analyzed for data extraction (Table 1) [73,74,75,76,77]. The PRISMA (Preferred Reporting Items for Systematic literature reviews and Meta-Analyses) 2020 guidelines were followed for this systematic literature review.

**Figure 1:**
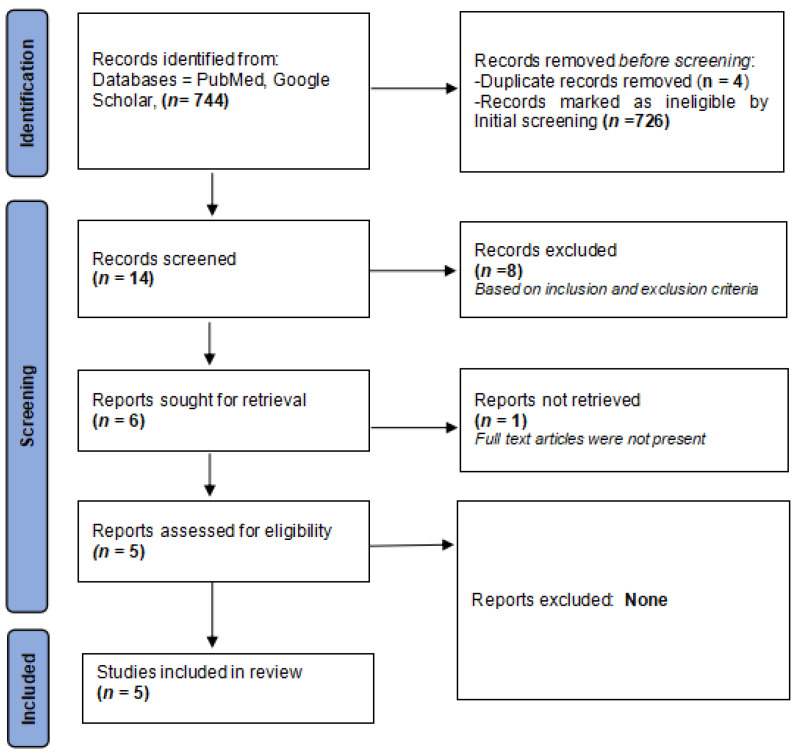
PRISMA flowchart detailing the search strategy.

Figure 1 displays a flowchart outlining the search strategy.

### 3.1. Study Characteristics 

The various characteristics of the included studies are summarized in Table 1 [73,74,75,76,77].

**Table 1:** Comprehensive summary of the included studies.

S. No.	Author(s)/Year/Country	Type of Study	Age/Sex/ Follow-Up	Sample Size	OLP Diagnosis	Treatment Plan	Test of Significance	Outcome	Conclusion
1.	Loré B et al., 2016 [73] Italy	Pilot study	8 males and 12 females, mean age of 56 years (range 40–74) completed the study with follow-up at 2, 4, 8, and 12 weeks.	20	Clinical and histopathological Diagnosis	OLP patients were divided into three groups. Reticular OLP patients were treated with cyclosporin mouth rinses OD for 8 weeks, Plaque-like OLP patients were treated with 0.05% retinoic acid lotion BID for 8 weeks, and erosive OLP patients were treated with PRP gel once a week for 8 weeks respectively. Evaluation for clinical improvement (complete response, partial response, and no response) was noted at 2, 4, 8, and 12 weeks.	Not Applicable	(I) Reticular (*n* = 10) Treatment-Cyclosporin Complete: 2 Partial: 5 No response: 3 (II) Plaque (*n* = 5) Treatment-Retinoic acid Complete: 3 Partial: 1 No response: 1 (III) Erosive (*n* = 5) Treatment-PRP Complete: 2 Partial: 2 No response: 1	OLP is associated with periods of remissions and exacerbations, hence, clinical management should be based on the clinical phenotype of OLP. Periodic follow-up with a detailed clinical examination is imperative.
2.	Ahuja US et al., 2020 [74] India	Prospective, case control, randomized clinical trial.	18 females & 2 males in the age range of 28–60 years (mean age 44.5 years); 4 months follow up	20	Clinical and histopathological Diagnosis	20 OLP patients were divided into 2 groups. 10 patients in each group were given weekly intralesional injections of corticosteroid and PRP respectively for 2 months. The patients were followed up for 4 months to evaluate pain/burning, erythema, and size of the lesion.	Unpaired *t*-test	Pain Scores: At the 4-month follow-up: NOT significant Lesion Size: At the 4-month follow-up: NOT significant Erythema scores: At the 4-month follow-up: NOT significant	The efficacy of intralesional PRP therapy was found to be similar to that of intralesional triamcinolone acetonide in the treatment of erosive OLP. Furthermore, PRP therapy exhibited less recurrence and no adverse effects.
3.	Shinnawi UE et al., 2021 [75] Egypt	Cohort Study	7 females & 3 males in the age range of 50–65 years	10	Clinical and histopathological Diagnosis	10 erosive OLP were given weekly intralesional PRP injections for 4 weeks. The patients were evaluated for pain (VAS) and the size of the lesion.	Friedman test and Wilcoxon test	Pain Reduction: At 4 weeks follow-up: Significant Clinical Scores: At 4 weeks follow-up: Significant	PRP injections exhibited significant efficacy in ameliorating the signs and symptoms in steroid-resistant erosive OLP cases.
4.	Hijazi AH et al., 2022 [76] Egypt	Pilot randomized controlled clinical trial	18 females & 2 males in the age range of 24–65 years	20	Clinical and histopathological Diagnosis	20 OLP patients were divided into 2 groups. 10 patients in each group were given weekly intralesional injections of PRP and corticosteroid respectively for a month.	Wilcoxon test	Pain Reduction: (I) At 4 weeks follow-up: Significant (II) At 3-month follow-up: Significant (III) At the end of the treatment: NOT significant Clinical Scores: (I) At 4 weeks follow-up: Significant (II) At 17 weeks follow-up: NOT significant (III) At end of treatment: NOT significant	Injectable PRP therapy may be regarded as an efficacious therapeutic regimen for erosive OLP cases.
5.	ElGhareeb MI et al., 2023 [77] Egypt	Case-control study	14 females & 10 males in the age range of 30–72 years; 3-month follow-up.	24	Clinical and histopathological Diagnosis	24 OLP patients were divided into 2 groups. 12 patients in each group were given intralesional injections of PRP and corticosteroid respectively every 2 weeks for 2 months.	Mann–Whitney test, Paired Wilcoxon Test and Chi-square test.	REU: (I) PRP (before) vs. Steroids (before): NOT significant (II) PRP (after) vs. Steroids (after): NOT significant (III) PRP (before) vs. PRP (after): Significant (IV) Steroids (before) vs. Steroids (after): Significant NRS: (I) NRS (before) vs. Steroids (before): NOT significant (II) NRS (after) vs. Steroids (after): NOT significant (III) NRS (before) vs. NRS (after): Significant Steroids (before) vs. Steroids (after): Significant	Injectable PRP therapy exhibited a safe therapeutic profile in OLP patients. However, intralesional PRP therapy was associated with more adverse effects (especially pain) and a higher relapse of OLP lesions after a 3-month follow-up.

Out of the five included studies (a total of 94 study participants), two studies each were cohort studies [73,75] and randomized clinical trials [74,76], respectively, and the fifth study was a case–control study [77]. Three studies were from Egypt [75,76,77], and one study each was from India [74] and Italy [73]. In all the studies, the diagnosis of OLP was made based on clinical and histopathological parameters [73,74,75,76,77].

All five studies in our systematic review included both genders, although females were primarily included [73,74,75,76,77]. Females (a total of 69 females) predominated the study population in contrast to males (25 males). The mean age of the study participants in all the included studies ranged between 40 and 60 years [73,74,75,76,77].

Three studies compared the efficacy of intralesional corticosteroid injections with PRP injections [73,76,77]. One of the studies evaluated the use of intralesional PRP injections in OLP patients recalcitrant to conventional steroid therapy [75]. Loré B et al. emphasized that clinical management should be based on the clinical phenotype of OLP. The study participants were recalcitrant to conventional corticosteroid therapy. They treated the reticular OLP cases with cyclosporine mouthwash, plaque-like OLP cases with 0.05% retinoic acid lotion, and erosive OLP cases with PRP gel [73].

### 3.2. Outcome Parameters

The patients were evaluated for a reduction in pain and clinical scores based on changes in the appearance and severity of the lesion.

Three included studies assessed the study outcomes based on pain diminution (VAS scores) and differences in the appearance and size of the lesion [74,75,76]. The VAS score was graded on a scale of 0 to 10, where 0 indicated no burning sensation and 10 indicated a severe burning sensation.

ElGhareeb MI et al. [77] used a different score for assessing pain. The Numeric Rating Scale (NRS) score that they used also evaluated the intensity of symptoms on a numerical scale ranging from 0 to 10, with 0 being no symptoms and 10 being the worst imaginable symptoms possible.

Loré B et al. [73] was the first study to evaluate the role of PRP therapy in the treatment of OLP. In their study, the focus was primarily on complete, partial, or no healing. They did not individually assess the intensity of pain or erythema.

The appearance and severity of the lesion were evaluated on a scoring system that differed in each study. Ahuja US et al. [74] employed the Thongprasom scale to assess the clinical appearance of the lesion and defined the appearance of erythema as a score of 1: mild erythema, 2: moderate erythema, and 3: severe erythema. The lesion size was scored as 0: normal mucosa, 1: size up to 0.25 cm^2^; 2: size up to 1 cm^2^; and 3: lesions > 1 cm^2^ area. After a gradual follow-up period of 4 months, both the steroids and PRP groups showed a decrease in the mean size of the lesion, but the comparative p-values were found to be insignificant.

A cohort study by Shinnawi UE et al. [75] evaluated the clinical appearance of lesions by using the Thongprasom scale. The scale decreased from the first day to the first week, but the difference was not significant. Further, the decrease in the Thongprasom scale between the second week and the third/fourth weeks was significant. However, the score was not significant between the third and fourth weeks.

Similarly, a randomized controlled clinical trial conducted by Hijazi AH et al. [76] also assessed the clinical picture by using the Thongprasom scale. At the end of the trial, they noticed a non-significant difference between the two groups of OLP, where one received PRP therapy (Group A) versus another that was treated with corticosteroids (triamcinolone acetonide injections—Group B). However, a significant statistical difference was observed in clinical scores between the two groups by week 4. Additionally, when comparing clinical scores in both Groups (A) and (B), there was no statistical difference at weeks 4 and 17. This indicates that PRP injections have a gradual and consistent clinical response when compared to corticosteroid injections.

ElGhareeb MI et al. [77] also divided their patients into two similar groups. Group A enrolled 12 patients treated using PRP therapy, while Group B had 12 patients treated with corticosteroid (triamcinolone acetonide) injections. Both groups were assessed for reticulation/keratosis, erythema, and ulceration (REU) scores and NRS scores for pain and clinical scores. Inter-group differences were not statistically significant when compared for REU and pain score (NRS) before or after treatment. On the contrary, intra-group differences before and after in each group were statistically significant. When finally evaluating the response of PRP therapy in patients, approximately 66.6% of patients showed a complete response. Interestingly, this study also highlighted that the number of side effects and chances of recurrence were also higher among those receiving PRP treatment.

The pioneering study conducted by Loré B et al. [73] enrolled patients into three groups. Reticular OLP patients were treated with cyclosporin mouth rinse OD for 8 weeks, plaque-like OLP patients were treated with 0.05% retinoic acid lotion BID for 8 weeks, and erosive OLP patients were treated with PRP gel once a week for 8 weeks. Clinical improvement in the lesions was evaluated at 2, 4, 8, and 12 weeks during patient follow-up. Out of all the patients, seven patients each reported a complete/partial response, whereas, six patients were non-responsive to therapy. The study emphasized that the management of OLP should be based on the clinical phenotype of OLP.

### 3.3. Assessment of Risk of Bias

The R-based Robvis software package was employed to evaluate the risk of publication bias. The majority of the domains were found to have a low risk of bias. Among the five studies included in the analysis, four studies (80%) demonstrated a low risk of bias, while one study (20%) had some concerns.

**Figure 2:**
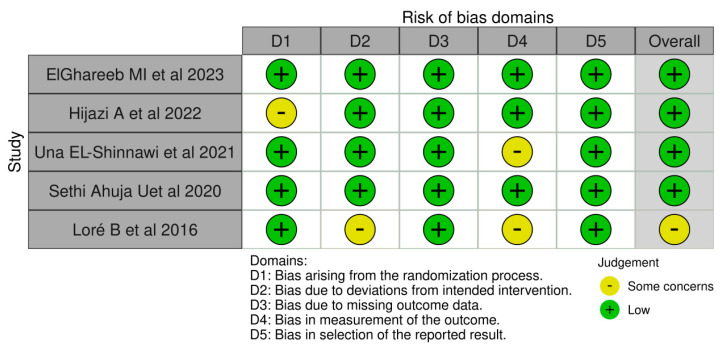
The risk of bias domains [73,74,75,76,77].

**Figure 3:**
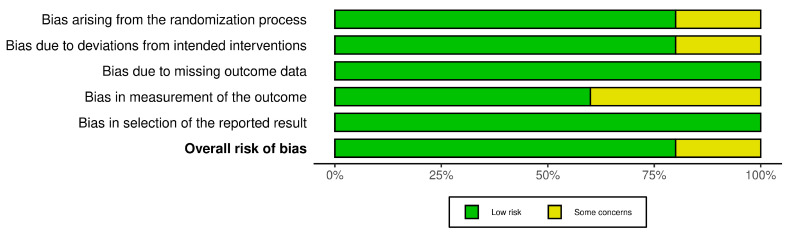
The overall risk of Bias.

Figure 2 and Figure 3 depict the degree of publication bias.

## 4. Discussion

OLP is a chronic inflammatory disorder, with an obscure etiology. It is considered autoimmune and characterized by T-lymphocytes targeted toward the basal layer of the oral epithelium [12].

The exact pathogenesis of OLP is still up for debate, but there is considerable evidence to suggest that immune dysregulation is a major factor. Immunopathogenesis could be related to several mechanisms, such as antigen-specific cell-mediated immune responses, non-specific mechanisms, autoimmune responses, and humoral immunity [77].

One hypothesis for the development of OLP is that T lymphocytes are activated by presenting antigens via major histocompatibility (MHC) molecules, leading to keratinocyte apoptosis. This suggests that immune dysregulation plays a crucial role in OLP pathogenesis [1,13,78]. Another mechanism, called a non-specific mechanism, involves the overexpression of matrix metalloproteinases (MMPs) and mast cell degranulation, which exacerbate T-cell accumulation, basement membrane destruction, and keratinocyte apoptosis [1,78].

Therefore, OLP is regarded as a chronic inflammatory mucosal disorder mediated by T lymphocytes. However, some researchers have proposed that autoimmunity may also contribute to OLP pathogenesis. This is based on the fact that CD8+ cytotoxic T lymphocytes are capable of recognizing antigens that are associated with major histocompatibility complex (MHC) class I on affected keratinocytes [1].

Osteopontin (OPN), CD44, and Survivin proteins play a role in OLP pathogenesis. OPN functions as an inflammatory cytokine and facilitates the migration and recruitment of macrophages and T lymphocytes [79]. CD44 is a primary glycoprotein receptor for OPN that mediates cellular attachment and chemotaxis and has a potential role in lymphocyte activation, proliferation, and migration [80].

Mucosal alterations in OLP patients may affect the homeostatic balance of oral epithelial cells, thus causing an altered equilibrium between cellular proliferation and cellular death (apoptosis) [81]. Survivin protein is crucial for cell survival, serving a dual purpose of regulating cell division and inhibiting apoptosis by interacting with various caspases [82].

Santarelli et al. demonstrated that OLP patients exhibit increased levels of osteopontin and CD44 and decreased levels of survivin. The study also demonstrated a corroboration between elevated osteopontin levels and elevated survivin levels to severe inflammation and minimal inflammation [83].

OLP is regarded as a multifactorial pathology with a plethora of triggering and exacerbating agents, including drugs (angiotensin-converting enzyme inhibitors, non-steroidal anti-inflammatory drugs), dental restorative materials (amalgam, composite resins), psychological stress, trauma (Koebner’s phenomenon), nutritional deficiencies (iron, B12, vitamin A, C, D, E, and B12 deficiency), viruses (hepatitis C virus, Epstein-Barr virus, varicella zoster virus), and genetic polymorphisms. OLP is also associated with systemic ailments (thyroid and liver dysfunction, hypertension, dyslipidemia) and autoimmune diseases (type 1 diabetes mellitus, Sjogren’s syndrome, systemic lupus erythematosus) [10,15].

The incidence of OLP is higher in females between the ages of 40 and 60 years, with a female-to-male ratio of 1.5:1 [13]. This higher prevalence of OLP in females may be due to their greater susceptibility to stress and hormonal imbalances [74].

Our study results were consistent with the existing literature. All five studies included in our systematic review enrolled participants of both genders, but females were predominant. In total, 69 females were included in the study population, compared to 25 males. The average age of study participants in all five studies ranged from 40 to 60 years [73,74,75,76,77].

A comprehensive medical history, thorough clinical and oral examination, and histopathological evaluation are often necessary to arrive at a definitive OLP diagnosis. However, when characteristic, bilaterally symmetrical, reticular oral lesions are present, a provisional clinical diagnosis may be sufficient [5]. Histopathological diagnosis can confirm the provisional clinical diagnosis and also help rule out cellular atypia and malignant changes [5,13].

In recent times, modern non-invasive methods, such as dermoscopy, optical coherence tomography, and reflectance confocal microscopy, have replaced traditional invasive diagnostic techniques for both diagnosis and therapeutic monitoring of OLP. These modern methods may aid in detecting the risk of malignant transformation and diagnosing oral squamous carcinoma earlier [84].

OLP was diagnosed based on the clinical and histopathological examination in all the included studies in our systematic review.

Corticosteroids are considered the primary treatment for OLP. Unfortunately, achieving complete remission and preventing disease relapse after discontinuing the medication are major challenges. Additionally, the viscoelastic properties of the oral mucosa make it difficult for topical pastes or gels to adhere and be absorbed before being rapidly cleared away [13,85].

OLP is characterized by cycles of exacerbation and remission, making it a persistent and prolonged condition. As a result, long-term steroid therapy is often utilized, but it can lead to several adverse effects. These can include local effects, such as oral candidiasis, altered taste, mucosal fragility, and drug reactions, as well as systemic effects, including adrenal suppression, hypertension, hyperglycemia, psychiatric issues, osteoporosis, and obesity. Furthermore, certain medical conditions, such as pregnancy, breastfeeding, diabetes mellitus, hypertension, herpetic infections, human immunodeficiency virus (HIV) infection, tuberculosis, and glaucoma, make systemic steroid therapy inappropriate. Therefore, there is a need for alternative treatment strategies for OLP [13,86].

Platelet-rich plasma (PRP) is an autologous blood product, which contains a high platelet concentration in a small amount of plasma [52]. The conception and explanation of PRP can be traced back to the field of hematology, where it was employed as a transfusion product in thrombocytopenic patients [87,88].

PRP has garnered increasing attention in recent years due to its possible applications in regenerative medicine, including cardiovascular surgery, dermatology, orthopedics, soft tissue repair (such as muscle, ligament, and tendon injuries), urology, cosmetics, and maxillofacial surgery [89,90]. The published literature has demonstrated promising results for the use of platelet concentrates in soft tissue healing, such as PRP-augmented bone grafts, in oral and maxillofacial surgery [91,92,93,94].

Platelet-rich plasma (PRP) is obtained from the patient’s blood through centrifugation, resulting in a concentrated mixture of growth factors (GFs) and cytokines. These bioactive factors can influence inflammation, cell proliferation, stem cell migration, and angiogenesis, thereby ameliorating the reparative and regenerative potential. GFs in PRP bind to their receptors (GFR) and induce protein kinase B (Akt) and extracellular regulated kinase (ERK) activity. The stimulation of Akt suppresses two pathways: (a) glycogen synthase kinase-3 beta (GSK3B) that promotes β-catenin degradation, and (b) Bcl-2-associated death promoter (BAD), which is accountable for inducing apoptosis [95].

The balance between intracellular levels of reactive oxygen species (ROS) and intracellular biochemical antioxidants is crucial for preventing cell damage in healthy cells. However, when this balance is disrupted, oxidative stress (OS) occurs [96]. OS can result from the decreased generation of antioxidants, weakened antagonistic effects on ROS, or increased ROS production due to external stimuli or certain conditions, exceeding the body’s compensatory ability to fight oxidative stress. This leads to a relatively excessive amount of ROS that cannot be eliminated by the cell, causing high OS in the body. This high OS can damage proteins, lipids, and DNA, leading to dysfunction in the cell [97]. Studies have found a correlation between oxidative stress (OS) and inflammation and the immune system in patients with OLP. Reactive oxygen species, oxidative damage, lipid peroxidation, and an imbalance in the antioxidant defense system are associated with the occurrence and development of OLP [98].

In vitro studies have demonstrated that PRP treatment can prevent oxidative damage by activating nuclear factor (derived-erythrocyte) type 2 (Nrf2), which, in turn, increases the signaling of antioxidant response elements [99].

The pathogenesis of OLP is influenced by various cellular events that are mediated by different cytokines. Tumor necrosis factor α and IL-1 play a significant role in disease progression. Additionally, recent research has linked other cytokines, including IL-4, which are secreted by type-2 helper T cells, to the pathogenesis of the disease [50]. Rhodus et al. demonstrated significantly higher levels of nuclear factor-kappa B (NF-κB) inflammatory cytokines (TNF-alpha, IL-1-alpha, IL-6, and IL-8) in OLP tissue transudates [100].

Hepatocyte growth factor (HGF) plays a critical role in the anti-inflammatory effect of PRP. This potent anti-inflammatory cytokine inhibits the NF-κB signaling mechanism, thereby reducing inflammation [101].

It has been established that PRP plays a crucial role in regulating tissue repair and reducing inflammatory damage. PRP promotes the production of anti-inflammatory cytokines, a type of biological substance that helps the activated macrophages regulate the effect of pro-inflammatory cytokines. Anti-inflammatory cytokines achieve this by interacting with soluble cytokine receptors and cytokine inhibitors, thus regulating inflammation.

IL-1 receptor antagonists, IL-4, IL-10, IL-11, and IL-13 are the most important anti-inflammatory cytokines. Cytokine receptors for TNF-α, IL-1, and IL-18 may also act as inhibitors for the pro-inflammatory activities of other proteins [102]. IL-10, an effective anti-inflammatory cytokine, functions by suppressing the generation of pro-inflammatory cytokines (IL-1, IL-6, and TNF-α) while facilitating the generation of anti-inflammatory agents [103].

Platelets in PRP may serve as a potential source of inflammatory mediators and regulators. Following incubation with polyacrylamide beads, platelets may release a host of anti-inflammatory cytokines. These may include the likes of IL-1 receptor antagonist (IL-1ra), soluble tumor necrosis factor (TNF) receptor (sTNF-R) I and II, IL-4, IL-10, IL-13, and interferon γ. More precisely, IL-1ra suppresses IL-1’s bioactivity by blocking its receptors. Meanwhile, sTNF-RI and RII can attach themselves to free TNFα, which curbs signal transduction. IL-4, IL-10, and IL-13 can promote the generation of IL-1ra and decrease the production of TNFα-induced prostaglandin E2. Interferon γ stimulates the production of IL-18-binding protein, which inhibits IL-18 production [104].

The platelet functions are not only confined to hemostasis but are also involved in the inflammation process. Platelets release various substances capable of modulating the inflammatory reaction by interacting with leukocytes and endothelial cells. Among the most prominent immunomodulators are transforming growth factor-beta (TGF-β), platelet-derived growth factor (PDGF), soluble ligand (sCD40L), and platelet factor 4 (PF4) [105].

TGF-β serves in the primary immunosuppression and differentiation of T-regulator cells (Treg), depending on TGF-β. This was observed in immune thrombocytopenia patients, where reduced T-reg cells and TGF-β levels were seen. However, upon treatment with therapies that boost platelet count (e.g., immunoglobulin, dexamethasone), a quantitative and functional revival of T-reg cells was noted, thus substantiating the aforementioned hypothesis [106]. In addition, activated platelets express CD154, which has an impact on the adaptive immune response. CD40L, present mainly on activated platelets and T cells, acts as a transmembrane protein with a significant function in both innate and adaptive immune systems. The soluble trimers of CD40L (sCD40L) may trigger various biological processes by binding to receptors on antigen-presenting cells [107].

The YPF4/CXCL4 protein is a member of the CXC chemokine family in humans. It attaches to heparin and is released from α-granules of activated platelets. The published literature has demonstrated that PF4/CXCL4 assists in T-cell trafficking and may also play a role in T-reg development [108,109]. According to Shi et al., PRP may have a significant immunological role in sustaining Th cell homeostasis and limiting Th17 cell development and response [110].

The efficiency of PRP in promoting wound healing and tissue regeneration is currently a topic of academic debate [51]. For more than three decades, PRP has been employed in tissue healing due to its capacity to repair tissues. The activation of platelets leads to the release of growth factors, such as PDGF, VEGF, EGF, IGF, TGF-β, and fibronectin, which continue to be released for up to 7–10 days following the topical application of PRP. This process promotes cell migration and proliferation, angiogenesis, and tissue regeneration [52]. It is thought that the ability of PRPs’ potential to accelerate the healing of both soft and hard tissues justifies their use in treating chronic oral diseases [53,54].

The workflow summarizing the use of PRP in OLP I is represented in Table 2 [52,53,54,99,100,101,102,103,104,105,106,107,108,109,110].

The validity and accuracy of both the visual analog scale (VAS) and numerical rating scale (NRS) have been substantiated in the OLP population through psychometric testing. The VAS pain scale, which consists of a 100 mm horizontal line labeled “no pain” at one end and “worst pain imaginable” at the other end, is used to measure pain intensity. The NRS pain scale measures the severity of oral pain a patient is currently experiencing on a scale of 0–10, using whole numbers (an 11-point scale) [111].

The NRS pain scale is considered to have better construct validity than the VAS due to its stronger correlation with clinical manifestations. Additionally, the NRS is simpler to score, easier for patients to comprehend and complete, and can be used for a wide range of patients (geriatric and those with fine motor neuron disabilities). These strengths make the NRS a superior instrument to the VAS for measuring oral symptoms in the OLP population [112].

However, in our systematic review, three studies evaluated pain and burning sensations on the visual analog scale (VAS) [74,75,76], and one study [77] used the NRS scale to assess pain.

The literature highlights the importance of having a global comprehensive scoring system for OLP patients that can lead to standardized outcome measures [13]. Thongprasom et al.’s clinical scoring system [113] is the most commonly employed, although other OLP scoring systems have also been recommended, including those by Chainani-Wu N et al. [111], Escudier M et al. [114], Piboonniyom S-O et al. [115], as well as the Reticulation–Erythema–Ulceration (REU) scoring system [115].

In our systematic review, three studies used the clinical scoring system from Thongprasom et al. [74,75,76], and one study used the REU scoring system [77]. However, Loré B et al. primarily focused on evaluating the complete, partial, or non-healing of OLP lesions and did not include individual assessments of pain intensity or erythema [73].

Recent studies have demonstrated that patients with OLP may have dysbiosis [9,116,117]. To maintain eubiosis, proactive measures, such as the use of paraprobiotics and postbiotics, may be effective. Paraprobiotics, a novel adjuvant therapeutic regimen for periodontal diseases, not only function as an effective regimen for maintaining oral health at home but also evaluate the cellular and inflammatory variables due to their immunomodulatory actions [118]. Further, Butera et al. employed a postbiotic-based gel containing lactoferrin and aloe barbadensis leaf juice powder as a treatment modality for periodontitis [119].

The significance of novel therapies in different dental domains is underscored by these recent therapeutic advancements. Nevertheless, further studies are necessary to enhance our understanding of the treatment modalities for OLP.

Our review had a few limitations. Firstly, the literature search was carried out on only two search engines, PubMed and Google Scholar. Secondly, all the studies included in our review had small sample sizes, which made it challenging to evaluate the therapeutic efficacy of PRP. Thirdly, the patient follow-up periods varied among the included studies. Moreover, there were variations in the method of PRP administration and the clinical type of OLP among the included studies. For instance, while three studies used intralesional PRP in erosive OLP [74,75,76], ELGhareeb et al. [77] used intralesional PRP in four erosive, two reticular, and six mixed OLP cases. Loré B et al. [73], on the other hand, used PRP gels in erosive OLP patients.

The studies included in our review displayed varying grades of heterogeneity, either clinically or statistically. Variations were observed in the participant number, study designs, treatment employed, and study results, which made it challenging to carry out a meta-analysis. Confounding factors (ethnic and demographic variations) and the technique used to obtain PRP further added to the limitations of our systematic review at the outcome level. As previously stated, the lack of a globally comprehensive scoring system for OLP also presented a challenge in standardizing outcome measures.

Although PRP is considered a safe treatment option with a scientifically understood mechanism of action and is relatively noninvasive, it is not approved by the Food and Drug Administration (FDA). Therefore, patients are offered this potentially beneficial treatment at a high cost, and it is not typically covered by insurance [66].

Additionally, evaluating PRP studies can be arduous because it is often used in conjunction with other therapeutic regimens, making it challenging to determine the efficacy of PRP alone. Recent insights and conflicting patient outcomes have raised questions about the clinical applications of PRP. One of the reasons for this may be the wide range of and variation in PRP and PRP-like systems, which differ in their collection volumes and preparation methods, and result in peculiar PRP characteristics and bioformulations. The lack of standardized PRP preparation protocols, coupled with inadequate reporting on bioformulations, further contributes to variable results [120].

PRP is generally considered a safe procedure, although there are some possible minor complications. These may include pain at the injection site, headaches, a feeling of heaviness in the head, swelling, urticarial rash as an allergic reaction, temporary skin discoloration, and bruising [66].

In our systematic review, a study by ElGhareeb et al. [77] reported that intralesional PRP therapy was associated with more adverse effects (especially pain) and a higher relapse of OLP lesions after a 3-month follow-up.

## 5. Conclusions

Due to the obscure etiopathogenesis and recalcitrant nature of OLP, there is currently no conclusive therapeutic protocol, despite careful efforts to establish one. Our study results demonstrated that PRP therapy resulted in a significant amelioration in objective and subjective symptoms in OLP patients, with minimal recurrences and adverse events. Nevertheless, it is imperative to conduct well-designed prospective clinical trials with large sample sizes to ascertain and substantiate the therapeutic role of PRP in OLP.

## Figures and Tables

**Table 2:** Comprehensive summary of the workflow of PRP in OLP.

S. No.	Salient Features	Mechanism
1.	Anti-oxidant and Anti-inflammatory	PRP treatment can prevent oxidative damage by activating nuclear factor (derived-erythrocyte) type-2 (Nrf2), which in turn increases the signaling of antioxidant response elements. Hepatocyte growth factor (HGF), a potent anti-inflammatory cytokine inhibits the NF-κB signaling mechanism, thereby reducing inflammation. Platelets in PRP may serve as a potential source of inflammatory mediators and regulators, and release a host of anti-inflammatory cytokines. e.g., IL-1 receptor antagonist (IL-1ra), soluble tumor necrosis factor (TNF) receptor (sTNF-R) I and II, IL-4, IL-10, IL-13, and interferon γ. IL-1ra suppresses IL-1’s bioactivity by blocking its receptors. sTNF-RI and RII can attach themselves to free TNFα, which curbs signal transduction. IL-4, IL-10, and IL-13 can promote the generation of IL-1ra and decrease the production of TNFα-induced prostaglandin E2. Interferon γ stimulates the production of IL-18-binding protein, which inhibits IL-18 production.
2.	Immunomodulatory	Platelets in PRP release various substances capable of modulating the inflammatory reaction by interacting with leukocytes and endothelial cells. Among the most prominent immunomodulators are transforming growth factor-beta (TGF-β), platelet-derived growth factor (PDGF), soluble ligand (sCD40L), and platelet factor 4 (PF4). TGF-β serves as the primary immunosuppressant and differentiation of T regulator cells (Treg) depend on TGF-β. CD40L, present mainly on activated platelets and T cells, acts as a transmembrane protein with a significant function in both innate and adaptive immune systems. Platelet factor 4 (PF4), a protein released from α-granules of activated platelets, assists in T cell trafficking, and may also play a role in Treg development. PRP may have a significant immunological role in sustaining Th cell homeostasis and limiting the Th17 cell development and response.
3.	Wound healing and tissue regeneration	Growth factors in PRP (PDGF, VEGF, EGF, IGF, TGF-β, and fibronectin) promotes cell migration and proliferation, angiogenesis, and tissue regeneration.

## Data Availability

Not applicable.
